# Full-Thickness Chest Wall Resection and Reconstruction for Locally Invasive Phyllodes Tumors: A Systematic Review

**DOI:** 10.3390/cancers17121907

**Published:** 2025-06-08

**Authors:** Yun Sun Lim, Ryan Tsui Hon Goh, Breanna Wei Ning Ang, Ailica Wan Xin Lee, Eugene Kwong Fei Leong, Lowell Leow, Qin Xiang Ng, Serene Si Ning Goh

**Affiliations:** 1 NUS Yong Loo Lin School of Medicine, National University of Singapore, Singapore 119077, Singapore; 2 Department of General Surgery, National University Hospital, Singapore 119074, Singapore; 3 Department of Cardiac, Thoracic and Vascular Surgery, National University Heart Centre Singapore, Singapore 119074, Singapore; 4 Saw Swee Hock School of Public Health, National University of Singapore and National University Health System, Singapore 119228, Singapore

**Keywords:** phyllodes tumor, chest wall resection, breast sarcoma, chest wall reconstruction, surgical oncology

## Abstract

**Background/Objectives:** Phyllodes tumors (PTs) are rare fibroepithelial breast neoplasms with a high propensity for local recurrence, particularly in borderline and malignant forms. Although wide local excision with negative margins is standard, the role of full-thickness chest wall resection (FTCWR) for PTs invading the chest wall remains unclear. This systematic review evaluated the outcomes of FTCWR in such cases, focusing on oncologic effectiveness and reconstruction. **Methods:** A comprehensive literature search of PubMed, Embase, Scopus databases, and Google Scholar (up to the end of November 2024) identified 18 case reports describing 18 patients (mean age of 42.9 years) with locally invasive PTs. **Results:** Most patients presented with large (>5 cm), recurrent malignant tumors fixed to the chest wall, with the largest measuring 38 cm. Median disease-free survival was 12 months (range: 1–60), with local recurrence in 5.6% and distant metastases in 22.2% of cases. Common complications included respiratory and wound-related issues. Despite the radical surgery, all patients achieved satisfactory cosmetic results. **Conclusions:** While FTCWR appears feasible and provides symptom relief and short-term disease control in select patients, the evidence remains limited to case reports. Its long-term oncologic benefit is still uncertain, and further research, including prospective studies and multi-center registries, is needed to elucidate its role in clinical practice.

## Simple Summary

Phyllodes tumors are rare fibroepithelial breast neoplasms that can occasionally present with aggressive local invasion, necessitating en bloc resection of the chest wall. This systematic review synthesizes the available literature on full-thickness chest wall resections performed for locally invasive phyllodes tumors, with a focus on surgical approaches, reconstructive strategies, and patient outcomes. A comprehensive search identified case reports and series documenting such interventions. We highlight the range of resection techniques employed, often involving ribs, intercostal muscles, and pleura, as well as common reconstructive options, including mesh repairs, muscle flaps, and prosthetic implants. Despite the radical nature of these procedures, most patients experienced good local control and acceptable functional outcomes. Postoperative complications were relatively infrequent and manageable. The findings underscore the need for a coordinated multidisciplinary approach in managing complex phyllodes tumors and they provide a reference for surgical decision-making and reconstructive planning in rare but challenging clinical scenarios.

## 1. Introduction

Phyllodes tumors (PT) of the breast, also known as cystosarcoma phyllodes, are rare fibroepithelial neoplasms, accounting for less than 1% of all breast neoplasms [1]. According to the World Health Organization (WHO), they are classified histologically as benign, borderline, or malignant [2]. While the majority are benign, approximately 10–15% exhibit malignant behavior [2,3]. Compared to most common types of breast tumors, rare breast tumors like PTs present unique diagnostic and therapeutic challenges. A hallmark challenge in managing PTs, particularly borderline and malignant subtypes, is their significant propensity for local recurrence (LR), with reported rates reaching up to 30% in malignant cases [4,5]. Conversely, distant metastases remain uncommon and are typically confined to malignant PTs, often involving the lungs, bones, or liver.

To minimize recurrence, the National Comprehensive Cancer Network (NCCN) [6] recommends wide local excision (WLE) with surgical margins of at least 1 cm for borderline and malignant PTs. Routine axillary staging is not indicated unless clinically suspicious lymphadenopathy is present, in which case targeted lymph node evaluation may be warranted. Total mastectomy is generally reserved for cases in which negative margins cannot be achieved through breast-conserving approaches. While this guideline-based approach is broadly accepted, some studies have advocated for a more aggressive surgical strategy in borderline and malignant PTs. In particular, mastectomy has been associated with lower recurrence rates compared to local excision, simple excision, or breast-conserving surgery in selected high-risk cases [7,8,9].

Complex chest wall invasion by PTs presents a rare but formidable surgical challenge, for which standardized management protocols are lacking. In patients with LR, surgical resection with wide, tumor-free margins remains the mainstay of management. However, the role of adjuvant therapies, including radiotherapy and chemotherapy, remains controversial, with limited evidence demonstrating improvements in recurrence or survival outcomes [10,11].

This review thus sought to address a clinical knowledge gap by synthesizing available evidence on the role of full-thickness chest wall resection (FTCWR) in achieving oncologic clearance for locally invasive PTs. We evaluated the oncologic outcomes, reconstructive strategies, postoperative complications, and patient-reported outcomes related to FTCWR for locally invasive PTs. The findings should help inform surgical decision-making, optimize patient selection, and guide future research in the management of this challenging clinical entity.

## 2. Methods

### 2.1. Study Protocol and Registration

This systematic review was conducted to evaluate the role of FTCWR in the management of locally invasive PTs. The review protocol was prospectively registered in PROSPERO (CRD42024621356). The methodology followed the Cochrane Handbook for Systematic Review of Interventions [12], and the reporting adhered to the PRISMA 2020 (Preferred Reporting Items for Systematic Reviews and Meta-Analyses) guidelines [13] to ensure transparency and rigor throughout the review and reporting process (completed PRISMA checklist can be found in Supplementary Table S1).

### 2.2. Search Strategy

A comprehensive search was conducted across PubMed, Embase, and Scopus from database inception to the end of November 2024. The search strategy combined relevant keywords and MeSH terms, including “full-thickness chest wall resection” and “phyllodes tumor”. No language or date restrictions were applied. The complete search strategy for the various databases is provided in Supplementary Table S2. Additionally, Google Scholar was used to search for grey literature, including case reports and institutional publications not indexed in traditional databases. Manual screening of reference lists and citation tracking of included studies were also performed to ensure comprehensive coverage.

### 2.3. Eligibility Criteria

In Covidence [14], studies were eligible for inclusion if they met the following criteria based on the PICOS framework:

- Population: patients diagnosed with locally invasive PT (primary or recurrent) confirmed by histopathology, with evidence of chest wall invasion on imaging or intra-operative findings.
- Intervention: FTCWR, defined as resection involving at least one rib and/or part of the sternum with overlying soft tissue.
- Comparators: not applicable.
- Outcomes: studies reporting at least one of the following: post-operative complications (immediate, early, or late), length of hospital stay, in-hospital mortality, duration of follow-up, LR, distant recurrence, survival or mortality outcomes, patient-reported outcomes, or quality of life (QoL).

Eligible study designs included case reports, case series, cohort studies, and randomized controlled trials, not limited to those in English, and published in peer-reviewed journals. Letters, editorials, commentaries (without original data), and conference abstracts and proceedings (without original data) were excluded.

### 2.4. Data Extraction and Synthesis

Data from the included studies were independently extracted by three reviewers (YSL, RTHG, BWNA) using a structured template to ensure consistency and completeness. Extracted study-level information included the first author’s name, publication year, and country of origin. Clinical data captured included age, gender, previous breast surgeries, presenting symptoms, radiological and histological findings, lesion status (primary or recurrent), details of surgical procedure, indications for reconstruction, any neoadjuvant therapies, specimen histopathology, reconstruction methods, flap types, prosthetic materials, surgical team, and adjuvant therapies.

Outcome data were categorized into three domains: (1) oncological outcomes: follow-up duration, disease-free survival (DFS), LR, distant metastases; (2) complications: postoperative complications, in-hospital mortality, reoperation rates; and (3) patient-reported outcomes and QoL. LR was defined as tumor reappearance at or near the primary surgical site (i.e., within the ipsilateral chest wall or breast region), while distant metastasis referred to tumor spread to anatomically remote sites such as the lungs, spine, or liver.

All data were tabulated in a structured charting matrix to facilitate comparison across studies. Data was analyzed and presented using descriptive statistics: medians for non-continuous variables, and percentages for dichotomous variables. The reviewers then collaboratively analyzed the data to identify recurrent patterns and themes, with iterative discussions involving senior authors (EKFL, SSNG) to refine the thematic framework. This thematic synthesis enabled the identification of key trends, variations in practice, and evidence gaps related to the use of FTCWR in managing locally invasive PTs.

### 2.5. Quality Assessment of Included Studies

Given the predominance of case reports and small case series, the methodological quality of the included studies was assessed using a modified version of the Newcastle–Ottawa Scale (NOS) [15,16]. Two independent reviewers (RTHG, BWNA) conducted the assessment across key domains: case selection, exposure and outcome ascertainment, causality, follow-up duration, and quality of reporting. A point was awarded for each criterion met. Based on total scores, studies were categorized as having low (5–6 points), moderate (3–4 points), or high (0–2 points) risk of bias. In addition, the quality of the grey literature was appraised using the AACODS checklist [17], which assesses six domains: Authority, Accuracy, Coverage, Objectivity, Date, and Significance. Similar to the NOS scoring system, grey literature sources were rated as low (5–6 points), moderate (3–4 points), or high (0–2 points) risk of bias. Any disagreements were resolved through consultation with a third reviewer (YSL or SSNG).

## 3. Results

### 3.1. Literature Retrieval

**Figure 1:**
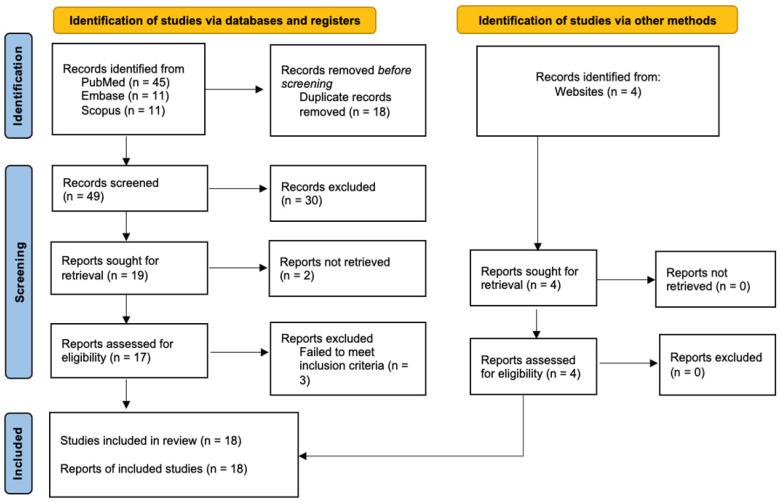
PRISMA flowchart showing the study selection process.

As illustrated in Figure 1, the initial literature search yielded 45 studies from PubMed, 11 from Embase, and 11 from SCOPUS. An additional four articles were identified from the grey literature. Of the 71 articles, 18 duplicates were removed. From the titles and abstracts of 53 non-duplicate studies identified, 30 articles were excluded. Subsequently, two reports could not be retrieved, and full-text screening of 21 articles excluded another three studies. Two did not involve chest wall invasion, and one revealed breast fibromatosis on histology. The final review included 16 case reports and two case series, each contributing one eligible patient, bringing the total number of patient data analyzed to 18.

The 18 studies [18,19,20,21,22,23,24,25,26,27,28,29,30,31,32,33,34,35] were from 12 different countries, with the majority from India (n = 5/18). Follow-up was reported in 17 out of 18 (94.4%) studies, and the median length of follow-up was nine months (range: 2 to 60 months). The evaluation of recurrence was based on clinical examination and radiological imaging.

### 3.2. Quality Assessment

The overall quality of the studies was good, with 72.2% of the studies rated as having a low risk of bias, and 27.8% having a moderate risk. For grey literature specifically, all four articles were rated as having a low risk of bias.

### 3.3. Patient and Tumor Characteristics Based on Included Studies

Table 1 presents a summary of the 18 studies and their results. The mean age was 42.9 years (range: 18–65). Of the 18 patients, 17 were female and 1 was male. Only five studies detailed the patients’ premorbid function; three of whom were pre-morbidly well, one had a previous history of methamphetamine abuse (but reported abstinence for over two years), whilst another had poor general health.

Most patients presented with a firm to hard fixed mass over the breast or chest wall, with large tumor sizes ranging from five cm to 38 cm, weighing up to 5.6 kg. A total of 17 patients had a unilateral lesion, and one had bilateral disease. Other findings included ulceration, necrosis, foul-smelling discharge, bleeding, pain, and infection. Four patients experienced rapid progression of the mass over a few weeks up to six months, and one experienced rapid recurrence within two months of a total mastectomy with free margins [25].

A total of 14 patients (n = 14/18, 77.8%) presented with recurrent disease, while two had a primary lesion (n = 2/18, 11.1%). The remaining two studies did not specify whether the lesion was primary or recurrent. At the time of presentation, eight (n = 8/14, 57.2%) had multiple (>1) recurrences. The median time to recurrence was 18 months, ranging from one month to 12 years.

For the 14 patients presenting with recurrence, 2 (n = 2/14, 14.3%) also had lung metastasis. All 14 were previously treated with WLE or mastectomies, with 3 (n = 3/14) reporting negative margins. However, five (n = 5/14, 35.7%) surgeons reported that their patients had likely been treated with inadequate or positive surgical margins on the previous resections and/or mastectomy. Previous diagnoses of recurrences include the following: four benign (n = 4/14, 28.6%), two borderline (n = 2/14, 14.3%), six malignant (n = 6/14, 42.9%) PTs, and two were undefined (n = 2/14, 14.3%). Adjuvant radiotherapy or chemotherapy was previously administered in five cases (n = 5/14, 35.7%).

### 3.4. Radiological Investigations

Pre-operative imaging indicating chest wall invasion was described in 16 studies (88.9%), with CT scans used in 14. MRI (n = 5), ultrasound (n = 2), and bone scintigraphy (n = 1) were also employed. Lung metastases were noted in two patients pre-operatively.

### 3.5. Histological Investigations

Of the studies reviewed, 17 studies reported the diagnosis of MPT and one of low-grade PT [18,19,20,21,22,23,24,25,26,27,28,29,31,32,33,34,35]. Of those reporting MPT, only 10 documented the pre-operative or post-operative histology (n = 10/17, 58.8%). Pre-operatively, 8 of 18 studies performed a biopsy diagnosing MPT. Only three specified the mode of biopsy; one incisional biopsy (n = 1/8), and two core biopsy (n = 2/8). Post-operatively, nine studies performed a histopathological examination on the resected specimen, with eight studies reporting MPT (n = 8/9) and one (n = 1/9) low-grade PT. Three (n = 3/17) studies presumed the diagnosis of MPT based on the histological findings from the previous surgery.

### 3.6. Operative Details of Full-Thickness Chest Wall Resection (FTCWR)

Table 2 presents a summary of the operative details of the surgery. A total of 11 cases were managed by a multidisciplinary team of surgeons (breast, thoracic, and plastic reconstructive surgery). Three involved only the thoracic surgeon, one involved only the plastic surgeon, one involved a single surgical oncologist, and two others did not specify.

Most surgeons completed the resection and reconstruction in a single session. Only Gupta et al. reported a two-staged procedure attributing to the extensiveness of the disease, initially performing FTCWR for the left chest wall tumor and debulking of the right chest wall tumor, followed by FTCWR for the right chest wall tumor [23]. Of the reported data, 15 surgeons had performed an en bloc resection, and one surgeon resected the tumor and chest wall separately. To establish adequate skin clearance circumferentially, some surgeons used an elliptical incision or Meyer’s skin incision technique. Mindikogˇlu et al. employed a Gigli saw to first excise the ulcerated mass at the skin level for better access to the chest wall [26]. Nagasaka et al. [28] and Suan et al. [33] proceeded with chest wall resection only after intra-operative confirmation of chest wall invasion of the tumor. Ito et al. [22] had an intra-operative frozen section analysis to confirm negative margins. Three studies reported the conduct of the surgery under general anesthesia, with two (n = 2/3) adding that the patient was placed in a lateral decubitus position.

**Figure 2:**
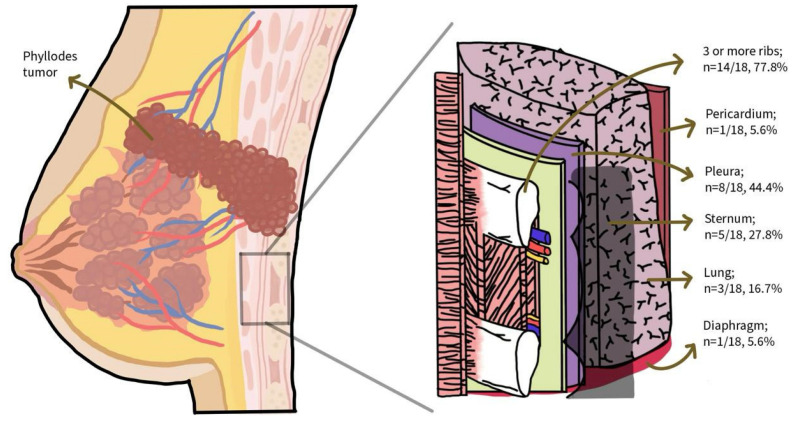
Schematic diagram of the anatomical layers of the chest wall involved in FTCWR.

Figure 2 shows the anatomical layers of the chest wall typically involved in a FTCWR. A total of 14 studies reported resection of three or more ribs (n = 14/18, 77.8%), five of the sternum (n = 5/18, 27.8%), eight of the pleura (n = 8/18, 44.4%), one of the pericardium (n = 1/18, 5.6%), one of the diaphragm (n = 1/18, 5.6%), and three of lung parenchyma (n = 3/18, 16.7%). Lung resection was indicated for metastasis, direct tumor invasion, and adherence to costal cartilage, with Schizas et al. describing the use of double lumen intubation to enable parenchymal resection in such cases [32]. Where there was invasion to a bipedicled DIEP flap, resection also involved the medial three-fourths of the flap [25]. Notably, 10 out of 18 studies reported achieving negative margins. Of these, five (n = 5/10) reported margins of at least 1.8 cm, and one reported a close negative margin of 3 mm (n = 1/10).

Adjuvant chemotherapy and radiotherapy were administered in one and four patients, respectively. Combined chemo-radiotherapy was administered in one patient.

### 3.7. Reconstructive Techniques

Reconstructive details reported by 17 out of 18 studies are reflected in Table 2. A total of 14 studies reported the combined use of both prosthetic material and soft tissue for the chest wall reconstruction (n = 14/17, 82.4%), two studies of only soft tissue (n = 2/17, 11.8%), and one study of autologous rib and soft tissue (n = 1/17, 5.9%). These can otherwise be categorized based on the rigidity of the material [36]: nine non-rigid, three rigid, and three combinations of non-rigid and rigid.

Of the eight studies reporting resection of the pleura, only Balachandran et al. had repaired the pleural defect with a prolene mesh [29]. Mindikogˇlu et al. repaired the pericardial defect by approximating it with a few sutures [26]. Gupta et al. repaired the diaphragm together with the anterior chest wall using a scaffold made from Steinmann pins and polypropylene mesh [23]. Ribs were reconstructed in two cases: one with a MatrixRIB system, and another with a composite pedicled autologous rib. Of note, the autologous rib was dissected such that it was incorporated with the flap and the lower two digitations of the left serratus anterior muscle.

The latissimus dorsi (LD) flap was most commonly used for soft tissue coverage (n = 10/18). Other flaps included rectus abdominis myocutaneous (n = 3/18), omentum, thigh, rotating cutaneous full-thickness, and combined bipedicled groin and LD flaps. In the case of the pedicled transverse rectus abdominis musculocutaneous (TRAM) flap, Küçükgüven et al. [24] described the anastomosis of the superficial inferior epigastric vein from the flap and cephalic vein from the right arm to enhance venous return on the basis of venous supercharging, in addition to the superior epigastric vessels of the pedicle. Tan et al. [34] described the use of two sets of recipient vessels, namely the thoracodorsal and thoracoacromial arteries to revascularize the combined musculocutaneous anterolateral thigh (ALT) with anteromedial thigh (AMT) flap, with venous supercharging facilitated by a long saphenous vein graft for the ALT. Fang et al. [25] described the challenge of a deficient recipient vessel for free flap reconstruction owing to tumor invasion of the right internal mammary vascular bundle, and prior use of the right lateral thoracic vascular bundle to supply the previous DIEP flap. The right thoracoacromial bundle was chosen over the left internal mammary vascular bundle due to the high risk of tumor invasion to the latter. Reconstruction with the ALT fasciocutaneous flap was successful, illustrating that the use of free flaps on two separate occasions can effectively address large soft tissue defects [25]. Boonipat et al. [35] described the need for a longitudinal skin paddle of 10–15 cm for the connection of the origin of the bipedicled groin and LD flap.

### 3.8. Post-Operative Outcomes and Complications

Most patients had a smooth recovery post-operatively. The most common complications were respiratory in nature (n = 4/18, 22.2%), including pneumonia, pleural effusion, and delayed expansion of the lung due to splinting from pain. These were successfully managed with antibiotics, drainage of the effusion by interventional radiology, and pain management, respectively. Mindikogˇlu et al. [26] described encountering a severe degree of paradoxical chest movement, critical enough to endanger the patient’s life, where spontaneous respiration started after the closure of the chest wall. This was managed with the application of a sponge pressure dressing so as to preserve the flap [26].

Complications associated with the reconstruction were reported in five cases. These included infection of the surgical site and mesh, as well as knee weakness as a result of sacrificing the vastus lateralis and rectus femoris muscles. These complications were successfully resolved with systemic antibiotics and local wound cleaning, and physiotherapy, respectively. Two patients experienced poor healing of skin grafts. Boonipat et al. [35] performed a repeat skin graft for the one spot that did not heal. On the other hand, Schizas et al. [32] described delayed wound healing with subsequent wound dehiscence and mesh contamination a month later, which required surgical intervention under general anesthesia to remove the mesh and restore the deficit with a TRAM flap.

Two studies briefly mentioned the patient’s post-operative pain experience. One was described by Suan et al. [33], as above, and Boonipat et al. [35] described local pain at the surgical sites, but neither documented their pain management regimen.

There were two inpatient mortalities related to FTCWR (n = 2/18, 11.1%). In the case reported by Gupta et al. [23], the patient received adjuvant chemotherapy (based on ifosfamide and epirubicin) and radiotherapy post-operatively. However, she passed on two months after completing adjuvant therapy, from an episode of pneumonia and respiratory failure. The second patient, as reported by Fang et al. [25], had a rapid progression of disease with distant metastasis to the lung, brain, and spine, leading to her demise two months later.

### 3.9. Oncologic Outcomes, Patient-Reported Outcomes, and QoL

From the reports, 15 out of the 18 patients were discharged safely. The median DFS was 12 months (range: 1–60 months). Four studies described positive patient-reported outcomes. Goel et al. [20] reported a decrease in the patient’s Hamilton Depression Rating Scale (HDRS) score from 24 to 3, where the patient “expressed her extreme happiness at getting rid of the foul-smelling tumor”. Rajesh et al. [31], Schizas et al. [32], and Boonipat et al. [35] all reported that FTCWR offered substantial emotional relief and an acceptable QoL for the patients.

Of the 17 studies with available follow-up data, the overall recurrence rate was 29.4% (n = 5/17), as summarized in Table 3. The median time-to-recurrence after FTCWR was 12 months. A total of 12 out of 17 patients did not have any further recurrence as of the last follow-up date (70.6%). Neto et al. [30] emphasized that their patient remained disease-free 48 months post-FTCWR, the longest period she has had without any recurrences. One reported disease progression of existing lung metastases [20].

Of the five cases reporting recurrence, one was an LR occurring 36 months from FTCWR (n = 1/5, 20%) [32], and four were distal recurrences occurring 1 [25], 2 [29], 12 [34] and 48 [18] months after FTCWR (n = 4/5, 80%). For the case of LR, it occurred inferior to the clavicle at a distance of five cm from the previous surgery. The patient was admitted for excision of the lesion and discharged, but a distant metastasis subsequently appeared 10 months later. There were further details on the management of this distal metastasis. Balachandran et al. [29] described disseminated metastases over two months post-operatively, to the ipsilateral axillary lymph nodes, bilateral lungs, spine, ribs, and other extra-thoracic sites (left supraclavicular, left anterior, and posterior chest walls). These were managed with palliative radiotherapy of 60 Gy in 30 fractions to the left axilla to prevent disease progression, radiotherapy of 20 Gy in five fractions to the thoracic spine (T4–T8), which successfully resolved her symptoms of lower limb weakness, and palliative chemotherapy (doxorubicin for six cycles, followed by one cycle of ifosfamide) after the recurrent lesion was deemed unresectable. However, she passed away three months after the onset of metastasis [29]. Fang et al. [25] described metastases to the left lung, brain, spine (T5–T6, L4), and right back muscle, with no mention of its management. Tan et al. [34] and Anile et al. [18] provided limited information on the distal recurrences and their management.

## 4. Discussion

This systematic review synthesizes 18 cases of FTCWR performed for locally invasive PTs [18,19,20,21,22,23,24,25,26,27,28,29,30,31,32,33,34,35], highlighting the rarity, complexity, and clinical challenges associated with such presentations. The predominance of malignant PTs (94.4%) and the high proportion of recurrent disease (77.8%) reflect a subgroup of patients with aggressive tumor biology, where conventional surgical strategies, such as WLE or mastectomy, have proven insufficient for long-term disease control. Furthermore, malignant PTs may exhibit satellite nodules or microscopic extensions beyond the gross tumor margin, further complicating the surgeon’s ability to achieve clear resection margins and potentially contributing to the high local recurrence rates reported in the literature. Our findings suggest that FTCWR, involving en bloc resection to achieve negative margins, can achieve short-term local control and symptom relief in selected patients, with a median DFS of 12 months and a recurrence rate of 29.4%. Notably, most recurrences post-FTCWR were distant metastases and uniformly fatal, underscoring the systemic nature of malignant PT once it breaches local boundaries and the need for early, aggressive intervention. These outcomes, while modest, are meaningful given the context of a heavily pre-treated, high-risk cohort, many of whom had multiple prior recurrences and presented with large tumors exceeding 5 cm [37,38,39]. These were also frequently associated with ulceration, necrosis, or rapid regrowth after prior surgical treatment. Although current NCCN guidelines emphasize margin-negative resection for borderline and malignant PTs, cases of chest wall invasion would necessitate radical surgery. FTCWR appears to fulfill this oncologic principle, offering a chance for local disease control where standard approaches fail. Yet, the persistently high rate of distant metastases highlights the limitations of surgery alone in altering the disease course for advanced PTs.

FTCWR also conferred substantial symptomatic relief and QoL benefits, particularly in addressing pain, ulceration, discharge, and malodor. Oncologic benefits were most apparent in younger patients without metastatic disease, though the procedure also offered palliative value for those with advanced disease. In patients with pre-existing metastases, long-term survival remains poor; however, FTCWR may provide significant palliative benefits, alleviating physical, psychological, and even spiritual suffering. As described by Goel et al. [20], one patient experienced profound emotional relief after surgery, despite eventual disease progression. Among the 27.8% of cases reporting QoL outcomes post-FTCWR, all documented improvement, highlighting the procedure’s role in supportive care and symptom control.

Complete surgical excision with margins of ≥1 cm remains the standard of care for borderline and malignant PTs. Despite the extensive nature of the surgery, most patients recovered well, with 83.3% of the patients discharged safely without major complications. In addition to surgery, adjuvant radiotherapy has been shown to reduce LR rates but does not significantly improve overall survival, according to current evidence [10,11,40]. This aligns with the findings in our review, where radiotherapy was inconsistently applied and appeared to offer limited benefit beyond local control, as also noted by Fang et al. [25]. The role of chemotherapy is even more uncertain, with minimal supporting evidence in the literature. Most studies, including [23], failed to demonstrate any clear survival benefit. Chemotherapy was rarely used and, consistent with the existing literature, showed no demonstrable impact on outcomes. Similarly, there is no evidence supporting the use of endocrine therapy. This reinforces the need for better systemic treatment strategies for malignant PTs, which remain lacking due to their rarity and the absence of prospective trials.

Chest wall reconstruction is critical in achieving optimal oncologic, functional, and aesthetic outcomes, particularly in cases of locally invasive malignant PTs. The principles of adequate rigid and soft tissue coverage guide reconstructive decisions. Various strategies exist, ranging from prosthetic materials to autologous tissue flaps, with the choice depending on defect size, location, and the need for structural support. Prosthetic meshes such as polypropylene, polytetrafluoroethylene (PTFE), or composite materials (e.g., methylmethacrylate sandwiched with mesh) conventionally provide rigid support for maintaining chest wall stability, preventing paradoxical respiration, and protecting underlying organs [41,42,43]. However, these materials carry risks of infection, foreign body reaction, and mesh extrusion. To mitigate these risks, biological reconstruction using muscle or myocutaneous flaps helps enhance vascular supply, improve wound healing, and provide durable coverage [42,43]. Studies have demonstrated that rigid prosthetic reconstruction combined with well-vascularized soft tissue flaps is associated with improved post-operative recovery and enhanced QoL [42,43,44].

The advent of titanium-based rib fixation systems, such as the MatrixRib, further strengthens stability, reducing post-operative pain and respiratory complications [43,45,46]. Hybrid reconstruction techniques incorporating both synthetic materials and allografts or homografts have demonstrated improved durability and reduced complications in extensive chest wall defects though supply and interim processing remain logistical challenges [43]. The use of porcine dermal collagen patches and bioengineered matrices as underlay to rigid reconstruction has gained traction due to their biocompatibility and reduced infection risk compared to conventional synthetic meshes [43,45,46,47]. The current evidence supports an individualized approach to reconstruction, considering defect location, extent of resection, and patient comorbidities [43,44,46]. In patients requiring total mastectomy and chest wall reconstruction, a combination of prosthetic and autologous tissue flaps has provided satisfactory long-term results. Considerations when choosing the method of reconstruction include the adequacy of coverage for the defect, the sufficiency of rigidity to prevent flail chest, and biological compatibility with the surrounding tissues. Future advancements including 3D-printed implants and tissue engineering technology could also further optimize durability and recovery [43,48,49].

Given the interruption of normal pulmonary physiology following FTCWR, the observed (22.2%) rate of pulmonary complications was unsurprising. Pulmonary complications include atelectasis, pneumonia, pneumonitis, and respiratory failure. Whilst advances in surgical technique, anesthesia, and perioperative care have helped mitigate some of these risks, as reported by Weyant et al. [50], the use of rigid prostheses, especially in patients with large chest wall defects, can support respiratory mechanics and potentially reduce the risks of pulmonary complications. Post-operative pain is another common concern that contributes to significant morbidity and negatively impacts both QoL and overall patient satisfaction. To mitigate post-operative pain and enhance recovery, Zacha et al. [51] reported favorable outcomes using intra-operative cryoablation of intercostal nerves and targeted local anesthesia to neuromuscular bundles, alongside conventional pharmacologic analgesia.

To further improve post-operative outcomes, a structured post-operative care plan would be helpful. Following Enhanced Recovery After Surgery (ERAS) protocols, early mobilization, ideally within the first 24 h, has been shown to reduce post-operative complications and healthcare costs [52,53]. During FTCWR, the sternum, pectoralis major, LD, and clavicles are often resected leading to destabilization of the shoulder and impairment of upper limb function. Targeted rehabilitation focusing on compensatory muscle groups, such as the teres major, deltoid, and triceps, can help reduce functional deficits. Additionally, as suggested by Sparreboom et al. [54], interventions such as incentive spirometry, chest physiotherapy, and inspiratory muscle training have been associated with shorter hospital stays and fewer long-term complications. Future investigation into prehabilitation, such as nutritional supplementation, personalized exercise programs, and smoking cessation programs, may improve postoperative outcomes and reduce complications after chest wall surgery.

### Strengths and Limitations of Review

This is the first systematic review to comprehensively evaluate the role of FTCWR in managing locally invasive PTs. A key strength lies in the comprehensive search strategy, incorporating both peer-reviewed studies and grey literature to capture a broad spectrum of clinical experiences. The use of established methodological frameworks, prospective protocol registration, standardized quality assessment tools, and adherence to PRISMA guidelines further enhance the rigor and transparency of this review.

Nonetheless, several limitations warrant consideration. The evidence base comprised only individual case reports, including single-patient data extracted from two case series, reflecting the rarity of FTCWR in locally invasive PTs. The small sample size and considerable heterogeneity in patient profiles, surgical approaches, reconstructive techniques, and outcome measures limit the ability to generalize the findings. The absence of comparative studies and long-term follow-up data also precludes definitive conclusions regarding oncologic control, optimal reconstruction strategies, and survival outcomes. Furthermore, inconsistencies in reporting key postoperative outcomes, such as pain control, functional recovery, and QoL, limit insights into patient-centered benefits. Variability in definitions of recurrence and complications across studies further complicates interpretation. These challenges reflect both the rarity of the condition and the complexities inherent in managing advanced PTs requiring FTCWR.

Future efforts should focus on establishing multi-center registries and standardized reporting protocols to facilitate the generation of higher-quality evidence. This would support clearer guidance on surgical decision-making, reconstructive planning, and long-term management in this rare but demanding clinical context.

## 5. Conclusions

While this systematic review suggests that FTCWR may be a feasible option for managing locally invasive PTs, the evidence is limited to isolated case reports. FTCWR appears to offer symptom relief and short-term disease control in selected cases; however, conclusions regarding its efficacy and long-term outcomes remain tentative. Further research, including prospective studies and multi-center registries, is needed to better define the role of FTCWR and confirm its long-term efficacy in this specific patient subgroup.

## Figures and Tables

**Table 1:** Summary of included cases (n = 18).

Study (First Author, Year, Country)	Age/Gender	Subtype	Tumor Status	Tumor Size (cm)	Chest Wall Invasion (Imaging Modality)	Type of Surgery	Post-Op Complications	Recurrence (Time, Site)	Mortality	Patient-Reported Outcomes
Anile, 2007, Italy [18]	65/F	Malignant	NR	>5	CT: chest wall invasion	FTCWR	NR	Distant ^1^ (48 mo DFS)	Yes	NR
Awwal, 2010, Bangladesh [19]	50/F	Malignant	Recurrent	>5	NR	En bloc FTCWR	NR	None	No	NR
Goel, 2018, India [20]	28/F	Malignant	Recurrent	15	CT: chest wall invasion + metastatic lung nodules	En bloc FTCWR	NR	None, but had progression of metastatic lung nodules	Yes (6 mo)	HDRS improved; 24 to 3
Chaudhry, 2013, Saudi Arabia [21]	30/F	Malignant	Recurrent	15	CT: chest wall invasion	En bloc FTCWR	NIL	None	No	NR
Ito, 2011, Japan [22]	39/F	Malignant	Recurrent	14	CT: muscle, sternum, and rib invasion + metastatic lung nodule	En bloc FTCWR	NIL	None	No	NR
Gupta, 2022, India [23]	NR	Malignant	Recurrent	18.5	CT: rib and costal cartilage invasion	2-staged FTCWR	Pneumonia	None	Yes (2 mo DFS)	NR
Küçükgüven, 2017, Turkey [24]	38/F	Malignant	Recurrent	11.7	US, CT, MRI: muscle invasion Bone scintigraphy: costochondral joint invasion	En bloc FTCWR	Pleural effusion	None	No	NR
Fang, 2019, Taiwan [25]	53/F	Malignant	Recurrent	>5	CT: DIEP flap and pleural invasion	En bloc FTCWR	NR	Distant (1 mo DFS)	Yes (3 mo)	NR
Mindikogˇlu, 1983, Turkey [26]	18/F	Malignant	Recurrent	25	CT: rib erosion	En bloc FTCWR	Paradoxical chest motion	None	No	NR
Murthy, 2014, India [27]	63/F	Malignant	NR	16	CT: muscle and rib invasion	En bloc FTCWR	NR	None	No	NR
Nagasaka, 1996, Japan [28]	45/F	Malignant	Primary	30	CT, MRI: chest wall and sternal invasion	En bloc FTCWR	Wound and mesh infection	None	No	NR
Balachandran, 2024, Malaysia [29]	20/F	Malignant	Recurrent	8.5	CT: muscle, ribs, and pleural invasion	En bloc FTCWR	NR	Distant (2 mo DFS)	Yes (5 mo)	NR
Neto, 2007, Brazil [30]	43/F	Low-grade	Recurrent	7	CT: chest wall invasion	En bloc FTCWR	NR	None	No	NR
Rajesh, 2017, India [31]	27/F	Malignant	Recurrent	18	MRI: Muscle and ribs invasion	En bloc FTCWR	NIL	None	No	Emotional respite
Schizas, 2021, Greece [32]	46/F	Malignant	Recurrent	26	CT, MRI: chest wall, costal cartilage invasion	En bloc FTCWR	Wound dehiscence and mesh contamination	Local (3 yr DFS)	No	Good QoL ^2^
Suan, 2023, Philippines [33]	45/M	Malignant	Primary	17.5	CT: chest wall, pleural, costal cartilage invasion	En bloc FTCWR	Pain, delayed lung expansion, poor healing of skin graft	None	No	NR
Tan, 2010, Singapore [34]	52/F	Malignant	Recurrent	12	NR	En bloc FTCWR	Knee weakness	Distant (12 mo DFS)	Yes (12 mo)	NR
Boonipat, 2019, USA [35]	43/F	Malignant	Recurrent	38	CT: rib invasion	En bloc FTCWR	Pain, poor healing of skin graft	None	No	Happy with recovery

Abbreviations: NR = not reported; CT = computed tomography; US = ultrasound; MRI = magnetic resonance imaging; DIEP = deep inferior epigastric artery perforator; DFS = disease-free survival, HDRS = Hamilton depression rating scale. ^1^ “Distant” recurrence refers to metastases (lung, spine, etc.). ^2^ QoL refers to any mention of patient satisfaction, symptom relief, or functional status.

**Table 2:** Operative details of FTCWR.

First Author, Year	No. of Ribs Resected	Sternum Resected	Pleura Resected	Pericardium Resected	Diaphragm Resected	Lung Resected	Margins	Reconstruction Method	Surgical Team
Anile, 2007 [18]	NR	NR	NR	NR	NR	NR	NR	Marlex mesh + LD flap	Thoracic surgeon
Awwal, 2010 [19]	NR	No	No	No	No	No	NR	Prolene mesh + LD flap	Plastic surgeon
Goel, 2018 [20]	NR	No	No	No	No	No	NR	LD flap	Multidisciplinary, including surgical oncologist
Chaudhry, 2013 [21]	≥3	Yes	No	Pericardial fat	No	No	Negative margins	PMMA cement marlex mesh sand-witch + LD flap	Breast, thoracic, plastic surgeons
Ito, 2011 [22]	4	Yes	No	No	No	Yes	2 cm negative margins	Composix mesh + LD flap	Breast, respiratory, plastic surgeons
Gupta, 2022 [23]	3 (Left side), 6 (Right side)	Yes	Yes	No	Yes	Yes	Negative margins	Polypropylene mesh + LD (Left) and greater omentum (Right) flap	Multidisciplinary (unspecified)
Küçükgüven, 2017 [24]	3	No	No	No	No	No	2 cm negative margins	Gore-Tex (2 mm) DualMesh + TRAM flap	Thoracic, plastic surgeons
Fang, 2019 [25]	3	No	Yes	No	No	No	2 cm negative margins	ALT flap	Thoracic, plastic surgeons
Mindikogˇlu, 1983 [26]	4	No	Yes	Yes	No	Yes	NR	Stainless steel mesh + LD flap	Thoracic, plastic surgeons
Murthy, 2014 [27]	3	No	No	No	No	No	NR	NR	NR
Nagasaka, 1996 [28]	3	Yes	No	No	No	No	NR	Marlex mesh + TRAM flap	
Balachandran, 2024 [29]	3	No	Yes	No	No	No	Close margin (3 mm)	Prolene mesh + MatrixRIB + LD flap	Breast, cardiothoracic surgeons
Neto, 2007 [30]	NR	No	No	No	No	No	4 cm negative margins	Composite mesh + PMMA cement + LD flap	Thoracic surgeon
Rajesh, 2017 [31]	4	Yes	Yes	No	No	No	1.8 cm negative margins	PTFE mesh + rotating cutaneous full-thickness flap	Surgical oncologist
Schizas, 2021 [32]	4	No	Yes	No	No	No	Negative margins	Polypropylene mesh + LD flap	Thoracic surgeon
Suan, 2023 [33]	5	No	No	No	No	No	Negative margins	Mesh + TRAM flap	Thoracic, plastic surgeons
Tan, 2010 [34]	6	No	Yes	No	No	No	NR	PMMA cement prolene mesh sand-witch + ALT and AMT flap	Thoracic, plastic surgeons
Boonipat, 2019 [35]	3	No	Yes	No	No	No	NR	Autologous rib + bipedicled groin and LD flap	Thoracic, plastic, general surgeons

Abbreviations: NR = not reported; LD flap = latissimus dorsi flap; TRAM flap = transverse rectus abdominis myocutaneous flap; ALT/AMT flap = anterolateral/anteromedial thigh flap; PMMA = poly(methyl methacrylate); PTFE = polytetrafluoroethylene.

**Table 3:** Characteristics of recurrent tumor cases after FTCWR.

Tumour Subtype	Age, Gender	Nature of Tumor (Before FTCWR)	Tumor Size (cm)	En bloc or Separately Resected	Margin Status	Margin Width (mm)	No. Ribs Resected	Pleural Involvement	Adjuvant Therapy
Malignant	46F	Fourth recurrence	18	En bloc	Negative	NR	4	Yes	NA
Malignant	65F	NR	>5	NR	NR	NR	NR	NR	NR
Malignant	53F	Second recurrence	>5	En bloc	Negative	20	3	Yes	Radiotherapy
Malignant	20F	Second recurrence	8.5	En bloc	Negative	3	3	Yes	NA
Malignant	52F	Second recurrence	12	En bloc	NR	NR	6	Yes	Radiotherapy

NA = not applicable; NR = not reported.

## Data Availability

The articles referenced in this manuscript are available from PubMed, Embase, and Scopus databases. No datasets were generated or analyzed during the current study.

## Supplementary Materials

The following supporting information can be downloaded at: https://www.mdpi.com/article/10.3390/cancers17121907/s1, Table S1: Full search strategy for the various databases; Table S2: Completed PRISMA checklist.
